# Binge alcohol and substance use across birth cohorts and the global financial crisis in the United States

**DOI:** 10.1371/journal.pone.0199741

**Published:** 2018-06-25

**Authors:** Justin Christopher Yang, Andres Roman-Urrestarazu, Carol Brayne

**Affiliations:** Institute of Public Health, University of Cambridge, Cambridge, United Kingdom; Universidad del Desarrollo, CHILE

## Abstract

**Background:**

The social and economic consequences of the global financial crisis (GFC) of 2007–9 has had serious impacts on population health, economic prospects, and overall wellbeing in all generations, particularly Millennials, Generation X, and Baby Boomers. The ways in which intergenerational inequality and global economic crises have affected population health, particularly with respect to excessive drinking and substance use in disadvantaged population groups has been understudied. Consequently, in this article, we seek to characterise the effects of the GFC on national trends in binge alcohol and substance use among Millennials, Generation X, and Baby Boomers. By doing so, we aim to contribute to a fuller understanding of the ways in which socioeconomic disadvantage engendered by the GFC has disparately affected the wellbeing of these generational cohorts.

**Methods and findings:**

We present results from National Survey on Drug Use and Health from 2007–16 to characterise binge alcohol and substance use among different generational cohorts in the United States during and after the GFC. Bivariate descriptive analysis and maximum-likelihood logit regressions focused on: (a) individual substances and binge drinking, (b) poly-use and (c) any use to simultaneously model how socioeconomic, demographic, and health characteristics were related to past-month substance use and to report the social, economic, and demographic correlates of substance use. Socioeconomic vulnerability was captured on a five-point scale comprised of: (1) health insurance status, (2) government assistance, (3) income, (4) self rated health, and (5) employment status. Millennials showed generally higher risk of binge alcohol and substance use during 2007–16 than Generation X, while Baby Boomers generally exhibited lower risk. Comparison of individual and poly-use patterns for the birth cohorts before and after reveals: Millennials were at significantly increased risk of use of binge alcohol (AOR = 1.51; 95% CI = 1.46–1.56), cocaine (AOR = 1.19; 95% CI = 1.03–1.37), heroin (AOR = 1.39; 95% CI = 1.01–1.91), and oxycontin (AOR = 2.33; 95% CI = 1.74–3.12) than Gen X while Baby Boomers were at significantly reduced risk of all substances. Nevertheless, Millennials were at significantly reduced risk of crack use (AOR = 0.33; 95% CI = 0.25–0.43) and poly-use (AOR = 0.56; 95% CI = 0.45–0.70) compared to Gen X. These differences may be related to measures of austerity and socioeconomic vulnerability. Millennials exhibited the highest vulnerability related to austerity with an average vulnerability score of 0.97 (95% CI = 0.96–0.98) while Baby Boomers exhibited the lowest average vulnerability score of 0.65 (95% CI = 0.64–0.66) with Generation X in between with 0.72 (95% CI = 0.71–0.73). Increased social and economic vulnerability after the 2007 crisis is strongly associated with higher rates of substance use in all generations.

**Conclusion:**

Millennials have been especially affected by socioeconomic changes associated with the GFC as reflected by their heightened vulnerability and increased use of binge alcohol and other substances compared to preceding generations. These findings suggest that attention is needed to address disparities in socioeconomic vulnerability, relationships to substance use and overall mental health of Millennials to mitigate the potential long term negative impacts of the GFC. In the context of a continuing international opioid and heroin crisis, the ways in which Millennials have been differentially affected warrants much greater attention both from policymakers and from researchers.

## Introduction

For Millennials, usually defined as those born after 1982 [1], the global financial crisis (GFC) of 2007–9 represents the most significant economic crisis of their lifetimes and the most serious economic crisis since 1929 with severe social and economic consequences that yielded a contraction of world output by 0.6% with potentially serious effects upon population health, economic prospects and overall wellbeing [2]. In the United States, the origins of the crisis were facilitated by low, introductory interest rates on sub-prime loans prior to 2007 [3]. As the Federal Reserve began to increase interest rates, the delinquency rate on home loans steadily increased which led to failures among American mortgage lenders and, eventually, major financial institutions, most notably Lehman Brothers [3]. What began in the United States as a subprime mortgage crisis escalated worldwide when ailing banks, severely exposed by the American crisis and at serious risk of defaulting, were bailed out and or nationalised by different governments in the EU which, in turn, led to rapid increases to sovereign debt obligations and consequent interventions on the part of the European Central Bank, the International Monetary Fund, and the European Commission [4]. Government responses to the GFC have been varied. In European countries such as the UK, Greece, Spain, Ireland, Portugal, governments engaged in austerity and fiscal consolidation programmes to cap the growth of public spending and to restore the trust of financial markets in European government bonds [5]. Consequently, many European countries saw major declines in public social spending, such as health and education [6]. In Greece, for instance, government financing of total inpatient expenditure in the health sector declined from 82.5% in 2009 to 75.2% in 2011 [7]. These changes in social spending have had profound collateral effects on poverty; for instance, material deprivation increased in the European Union from 9.1% in 2007 to 9.9% in 2012 [5]. In Greece, Spain, Italy, and Portugal, Matsaganis and Leventi note that some groups, especially the young and the poor, suffered cuts to both social programme funding as well as wages, while other groups, such as the elderly, only suffered minor losses to material wealth signaling a trend that became common in the retreat of social welfare across European countries [8].

In the United States, the American government, in contrast to their European counterparts with the exception of the UK, used austerity as the policy of choice to deal with the subprime crisis, but also engaged in a policy of fiscal stimulus through the *American Recovery and Reinvestment Act of 2009* (ARRA). Despite ARRA’s intended effects, the median family income in America dropped by about 8% [9], the 90/10 ratio, a measure of income inequality, increased by 11% [10], and jobs declined by 4.2% from 2007–9 [11]. In addition, quantitative easing, a policy meant to mitigate some of the effects of the GFC by increasing the size of the Federal Reserve’s balance sheet from less than $1 Trillion in 2007 to over $4 Trillion in 2015, was linked to increased income inequality as wealth among the middle and lower classes stagnated and declined with disproportionate effects on the young with control of 40 percent of the American GDP by the top 1% [12]. Economic inequality and hardship engendered by the GFC and its consequent effects on unfavourable labour markets have particularly affected Millennials [13]. In the United States, major labour trends such as the increase in temporary contingent jobs and the increase in overseas outsourcing have affected the entry of Millennials into the professional job ladder [14]. For those entering the job market in the United States, rising underemployment is as high as one in two among recent graduates [15] with wage inequality [14] becoming the *status quo*. In response, Millennials have sought ever higher educational attainment [16] with over 21 million students enrolled in higher education in 2010 [17], accompanied by increasing levels of indebtedness [18], partly due to increased reliance on student loans as a financing mechanism for higher education in the United States, now $1.4 Trillion or 7.5% of the USA GDP [19].

There is a growing focus on how intergenerational inequality and global economic crises have affected population health, particularly with respect to mental health in disadvantaged population groups [20], including research into excessive drinking [21, 22] and substance use [23]. Bor’s study of Americans has found, for example, that the prevalence of binge drinking has increased from 4.8% in 2006–7 to 5.1% in 2008–9, corresponding to a national increase of 770,000 binge drinkers in a 12 month period [24]. The ways in which intergenerational inequality and the GFC have impacted the mental health of Americans have been relatively understudied and pose a crucial question about how economic crises could differentially affect mental health and substance use patterns of different generations. In the analysis presented here, we present results from National Survey on Drug Use and Health (NSDUH) from 2007–16 to describe patterns of binge alcohol and substance use among different generational cohorts in the United States during and after the subprime mortgage crisis of 2007–8 with a focus on the socially and economically vulnerable. Our aim is to see how the behaviours of Millennials, Gen X and Baby Boomers have or have not been affected by the GFC. We examine rates of binge alcohol and substance use, as well as characterising the ways in which ethnicity and social and economic vulnerability are related to patterns of binge drinking and substance use.

## Methods

### Sample and procedures

Data for adults aged 18 and over were included from the 2007–16 cohorts of the National Survey on Drug Use and Health (NSDUH), an annual, nationally representative survey of civilian, non-institutionalised individuals aged 12 and above in the United States which measures the prevalence and correlates of substance use and self rated health (*N* = 307,935) [25]. Household selection for each year’s survey is conducted independently in all 50 states and the District of Columbia and excluded individuals with no fixed household address, active duty military personnel, and individuals living in institutionalised group quarters [25]. The survey is administered *via* face-to-face interviews using computer-assisted interviewing (CAI) to increase respondents’ cooperation and willingness to report honestly about topics such as illicit drug use behavior and mental health issues [25]. Questions pertaining to the use of regulated substances are self-administered [25]. Sampling and analytical weights are calculated based on population estimates from the 2000 or 2010 decennial census and provided with the NSDUH dataset to address unit- and individual-level non-response [25]. Respondents are given a $30 cash incentive following completion of the interview [25].

As the NSDUH is a publicly available dataset, this study was not considered human subjects research under the federal Common Rule, 45 CFR Part 46.

### Variables

#### Generational cohorts

Drawing upon Eyerman and Turner’s definition of generation, we categorised individuals from the NSDUH according to socially and culturally defined three groups, namely, Millennials, Generation X (“Gen X”) and Baby Boomers [26]. Cohort assignment was based on age at the time of each survey where (a) respondents aged 18 to 34 were classified as Millennials, (b) respondents aged 35 to 49 were classified as Gen X, and (c) respondents 50 or older were classified as Baby Boomers.

#### Social characteristics

Sex was coded as either female or male. Respondent ethnicity was coded as White, black, native American, Asian or Pacific Islander, Hispanic or Other. Education was coded such an education level of seventh grade or less were categorised as elementary, an education level between eighth grade and twelfth grade was categorised as secondary, and an education level higher than twelfth grade was categorised as tertiary. Marital status was coded as single, married, widowed, and divorced or separated. Metropolitan size was coded as large, small, or non-metropolitan based on 2010 census data and 2009 Core Based Statistical Area classifications provided by the Office of Management and Budget (OMB) [25].

#### Economic characteristics

Annual income was categorised as less than $20,000, between $20,000 and $49,999, between $50,000 and $74,999, and greater than $75,000. Poverty level, based on income as a percentage of the Federal Poverty Level (FPL), was categorised as either living in poverty (<100% FPL), income up to 200% FPL, and income above 200% FPL. A dichotomous variable was created to indicate whether a respondent received any form of government assistance (i.e. Supplemental Security Income, food stamps, cash assistance, and/or non-cash assistance).

#### Health and insurance characteristics

Self reported health was categorised as excellent, very good, good, or poor. The respondent’s primary health insurer was coded as private, Medicare, Medicaid, Tricare & Veterans Administration (VA), other, or uninsured.

#### Past-month substance use

Measures of past-month substance use were obtained for binge alcohol, cocaine, crack, heroin, recreational use of oxycontin (*i*.*e*. non-prescription), and methamphetamine. We recoded these variables as dichotomous variables indicating past-month substance use. In addition, we created two new variables to indicate cases of poly-substance use other than binge drinking when individuals responded positively to having used (a) more than one substance and (b) any use of a substance within the past-month. From 2015–16, the NSDUH surveys did capture data regarding past-month oxycontin use; consequently, for these years, we do not report past-month oxycontin use.

#### Social and economic vulnerability

We defined a new composite variable to measure social and economic vulnerability on a five-point scale. The index is a composite of multiple quantitative indicators of social and health vulnerability that by aggregating data in a continuous form delivers a single numerical result for each participant of the NSDUH. Through such an index, diverse issues can be combined into a standardised framework making comparisons possible. One point was given for each of (a) uninsured or insured on Medicaid, (b) government assistance recipient, (c) annual household income less than $20,000, (d) poor self rated health, and (e) unemployment. Consequently, we adjudicated a score of 0 to indicate the least vulnerable while a score of 5 to indicate the most vulnerable, based on the criteria described above. To our knowledge, this is the first attempt to develop an index with respect to social and economic vulnerability when analyzing substance use in the USA though vulnerability as a construct is accepted as a construct in other population analyses [27–30].

#### Imputed data

The NSDUH addresses item nonresponse using an imputation method known as predictive mean neighborhood (PMN) which has been applied to NSDUH datasets since 1999 [25]. PMN is applied in a stepwise fashion: (a) response propensity adjustment; (b) prediction modelling; and (c) hot-deck imputation [25]. In the NSDUH data imputation was used extensively for variables pertaining to ethnicity and government assistance and slightly less for education, marital status, income, and health insurer [25].

### Statistical analyses

All statistical analyses were performed in Stata 14. The NSDUH has a cross-sectional survey design with data collection each year conducted independently. Consequently, datasets from individual years were combined into a single file for analysis to allow for comparison between years. As our outcome variables of interest were dichotomous, we utilised bivariate descriptive analysis and multiple maximum-likelihood logit regressions with weighted least squares on (a) individual substances and binge drinking, (b) poly substance use and (c) any use to simultaneously model how socioeconomic, demographic, and health characteristics were related to past-month substance use for binge either alcohol, cocaine, crack, heroin, oxycontin, and methamphetamine a combination of any of these or any use of them. In our logistic regressions, Generation X served as the reference group against which Millennials and Baby Boomers were compared. Unadjusted and adjusted odds ratios (OR) with 95% CI’s were calculated for the odds of past month substance use and binge drinking. Multiple logistic regression analyses were done for past-month use of each of binge alcohol, cocaine, crack, heroin, oxycontin, and methamphetamine, separately adjusting for generational cohort, year, sex, ethnicity, level of education, marital status, self reported health, metropolitan size, type of health insurance, government assistance status, income, and income as a percentage of FPL. All analyses were weighted to account for the complex survey design of the NSDUH using analytical weights provided with each dataset.

## Results

The descriptive characteristics of our sample are shown in Table 1.

**Table 1:** Sample characteristics for adults age 18 and over by percentage (%), 2007–16.

Characteristic	2007(*n* = 37,708)	2008(*n* = 37,504)	2009(*n* = 37,707)	2010(*n* = 38,919)	2011(*n* = 39,133)	2012(*n* = 37,869)	2013(*n* = 37,424)	2014(*n* = 41,671)	2015(*n* = 43,561)	2016(*n* = 42,625)
Age Cohort										
Millennial	30.56(29.71–31.27)	30.49(30–31.44)	30.72(29.99–31.61)	30.79(29.59–31.22)	30.4(29.65–31.18)	30.41(29.51–31.25)	30.37(29.72–30.9)	30.31(29.36–30.97)	30.16(29.36–30.67)	30.01(30.14–30.7)
Gen X	29.34(28–29.39)	28.69(27.28–29.08)	28.17(26.63–28.06)	27.34(25.64–27.09)	26.36(25.09–26.7)	25.89(24.79–26.44)	25.61(24.41–25.82)	25.11(24.3–25.49)	24.89(24.23–25.46)	24.84(26.34–26.82)
Baby Boomer	40.1(39.86–41.79)	40.82(40.03–42.2)	41.11(40.89–42.86)	41.87(42.16–44.34)	43.24(42.6–44.81)	43.7(42.86–45.19)	44.02(43.7–45.46)	44.58(44.01–45.88)	44.95(44.26–46.05)	45.15(42.64–43.38)
Sex										
Male	48.24(47.4–49.07)	48.26(47.33–49.2)	48.29(47.35–49.23)	48.4(47.5–49.31)	48.09(47.25–48.92)	48.13(47.26–48.99)	48.17(47.32–49.03)	48.18(47.57–48.78)	48.21(47.46–48.95)	48.23(47.55–48.9)
Female	51.76(50.93–52.6)	51.74(50.8–52.67)	51.71(50.77–52.65)	51.6(50.69–52.5)	51.91(51.08–52.75)	51.87(51.01–52.74)	51.83(50.97–52.68)	51.82(51.22–52.43)	51.79(51.05–52.54)	51.77(51.1–52.45)
Ethnicity										
White	69.18(68.13–70.21)	68.8(67.87–69.7)	68.4(67.43–69.35)	68.02(67.18–68.86)	66.74(65.86–67.6)	66.28(65.23–67.32)	65.83(64.74–66.91)	65.25(64.18–66.3)	64.73(63.74–65.71)	64.39(63.46–65.32)
Black	11.4(10.69–12.16)	11.46(10.85–12.1)	11.6(10.95–12.29)	11.63(11.04–12.25)	11.5(10.91–12.11)	11.56(10.85–12.3)	11.7(10.89–12.57)	11.74(11.09–12.41)	11.78(11.19–12.4)	11.79(11.21–12.41)
Native American	13.29(12.53–14.09)	13.49(12.88–14.12)	13.7(12.97–14.48)	13.9(13.33–14.48)	14.61(13.88–15.37)	14.8(14.11–15.53)	15.03(14.4–15.68)	15.33(14.52–16.17)	15.58(14.93–16.25)	15.74(15.15–16.36)
Asian & Pacific Islander	4.57(4.152–5.028)	4.69(4.233–5.196)	4.75(4.217–5.356)	4.83(4.377–5.326)	5.24(4.699–5.849)	5.34(4.855–5.877)	5.41(4.823–6.071)	5.64(5.16–6.168)	5.75(5.292–6.253)	5.83(5.406–6.294)
Hispanic	0.49(.3756 –.6385)	0.43(.3564 –.5286)	0.47(.3651 –.6133)	0.48(.3904 –.601)	0.54(.4523 –.6516)	0.56(.4499 –.7086)	0.52(.4329 –.6289)	0.51(.4163 –.6136)	0.52(.4236 –.6343)	0.57(.4692 –.6861)
Other	1.07(.9209–1.236)	1.13(.9603–1.333)	1.07(.9276–1.223)	1.14(.9554–1.35)	1.37(1.219–1.544)	1.45(1.215–1.725)	1.5(1.298–1.733)	1.54(1.419–1.673)	1.64(1.484–1.807)	1.67(1.524–1.819)
Employment										
Full–Time	54.54(53.66–55.43)	54.17(53.13–55.22)	50.58(49.66–51.49)	49.87(48.88–50.86)	49.76(48.78–50.73)	50.01(49.17–50.85)	50.05(49.09–51.02)	51.17(50.44–51.91)	48.81(48.1–49.52)	49.23(48.47–49.99)
Part–Time	13.26(12.67–13.87)	13.56(13.01–14.13)	13.88(13.31–14.48)	14.44(13.89–15)	13.96(13.43–14.5)	13.97(13.5–14.46)	14.24(13.61–14.9)	13.75(13.32–14.2)	13.37(12.96–13.79)	13.1(12.56–13.66)
Unemployed	3.23(3.019–3.447)	3.96(3.62–4.332)	6.58(6.237–6.939)	6.47(6.115–6.837)	5.83(5.506–6.178)	5.74(5.371–6.135)	4.99(4.677–5.328)	4.51(4.255–4.788)	4.71(4.46–4.974)	4.56(4.291–4.842)
Other (including not in labor force)	28.97(28.17–29.79)	28.31(27.37–29.26)	28.96(27.99–29.95)	29.23(28.27–30.21)	30.46(29.39–31.54)	30.28(29.36–31.2)	30.71(29.82–31.61)	30.56(29.84–31.29)	33.11(32.27–33.96)	33.11(32.48–33.75)
Education										
Elementary	3.94(3.47–4.468)	3.52(3.173–3.903)	3.02(2.755–3.305)	3.37(3.013–3.759)	3.03(2.745–3.333)	3.06(2.744–3.418)	3.16(2.786–3.58)	3.23(2.953–3.526)	3.02(2.685–3.391)	2.99(2.75–3.242)
Secondary	43(42.11–43.89)	43.12(42.15–44.1)	43(41.85–44.16)	42.16(41.2–43.13)	41.13(40.25–42.02)	41.15(40.35–41.96)	39.85(38.81–40.89)	38.87(38.01–39.75)	36.51(35.66–37.37)	35.19(34.53–35.85)
Tertiary	53.06(52.18–53.94)	53.36(52.36–54.36)	53.98(52.87–55.08)	54.47(53.48–55.45)	55.84(54.95–56.73)	55.78(55–56.57)	56.99(55.9–58.08)	57.9(56.97–58.83)	60.47(59.59–61.35)	61.83(61.14–62.51)
Income										
Less than 20,000	18.27(17.49–19.08)	16.97(16.3–17.67)	17.63(16.88–18.41)	18.73(17.88–19.61)	19.33(18.6–20.08)	18.91(18.22–19.62)	18.38(17.53–19.27)	18.22(17.6–18.86)	17.85(17.28–18.44)	16.99(16.33–17.66)
20,000 to 49,999	33.11(32.14–34.08)	32.54(31.68–33.41)	32.8(31.98–33.64)	33.47(32.53–34.42)	32.49(31.73–33.25)	32.95(32–33.91)	31.48(30.66–32.32)	31(30.09–31.92)	30.02(29.27–30.79)	29.99(29.27–30.71)
50,000 to 74,999	18.27(17.39–19.19)	18.54(17.84–19.27)	17.2(16.56–17.85)	16.85(16.2–17.52)	17.09(16.48–17.72)	16.56(15.87–17.27)	17.17(16.38–17.99)	16.66(16.06–17.28)	16.65(16.09–17.23)	15.93(15.29–16.59)
More than 75,000	30.35(29.41–31.31)	31.95(30.95–32.96)	32.37(31.21–33.55)	30.95(29.84–32.08)	31.09(30.17–32.02)	31.58(30.46–32.72)	32.97(31.71–34.24)	34.12(33.19–35.06)	35.48(34.58–36.38)	37.09(36.12–38.07)
Marital Status										
Single	25.28(24.51–26.06)	26.07(25.39–26.77)	26.66(25.94–27.4)	26.7(25.89–27.52)	26.69(25.92–27.47)	27.1(26.32–27.9)	27.61(26.74–28.49)	27.95(27.23–28.68)	27.11(26.55–27.68)	28.5(27.75–29.27)
Married	55.4(54.51–56.27)	55.03(54.2–55.86)	54.56(53.45–55.66)	52.66(51.7–53.63)	52.88(51.86–53.9)	52.64(51.65–53.62)	52.12(51.09–53.14)	51.91(51.09–52.73)	52.72(51.96–53.47)	51.69(50.87–52.51)
Widowed	5.98(5.473–6.539)	6.02(5.595–6.464)	5.74(5.3–6.212)	6.41(5.922–6.942)	5.98(5.546–6.446)	6(5.492–6.56)	6.12(5.547–6.752)	5.77(5.432–6.135)	6.24(5.858–6.639)	5.9(5.485–6.34)
Divorced or Separated	13.35(12.74–13.98)	12.88(12.21–13.58)	13.04(12.42–13.69)	14.23(13.59–14.9)	14.45(13.81–15.12)	14.26(13.71–14.82)	14.15(13.6–14.72)	14.37(13.88–14.86)	13.94(13.4–14.5)	13.9(13.39–14.44)
Self Rated Health										
Excellent	23.24(22.59–23.9)	23.18(22.4–23.98)	23.47(22.74–24.21)	22.95(22.21–23.71)	23.16(22.41–23.92)	21.7(20.93–22.49)	22.25(21.52–23.01)	21.4(20.84–21.98)	21.68(21.02–22.34)	21.05(20.51–21.61)
Very Good	36.07(35.26–36.88)	35.84(34.79–36.91)	36.2(35.35–37.06)	36.59(35.67–37.53)	36.11(35.39–36.83)	36.43(35.65–37.21)	36.24(35.53–36.96)	35.94(35.23–36.65)	35.12(34.37–35.89)	35.83(35.13–36.53)
Good	27.08(26.27–27.9)	27.53(26.69–28.39)	27.54(26.65–28.45)	26.88(26.01–27.77)	27.01(26.38–27.64)	28.31(27.51–29.12)	28.04(27.33–28.76)	28.68(28.03–29.33)	29.25(28.5–30.01)	29.16(28.43–29.9)
Poor	13.62(12.85–14.43)	13.45(12.69–14.24)	12.79(12.19–13.41)	13.58(12.82–14.38)	13.73(13.11–14.36)	13.57(12.84–14.33)	13.46(12.7–14.26)	13.98(13.52–14.46)	13.95(13.38–14.54)	13.96(13.38–14.56)
Metropolitan Statistical Area Size										
Large Metropolitan	53.35(52.18–54.52)	53.25(52.16–54.35)	53.06(51.86–54.26)	53.56(52.37–54.75)	53.16(52–54.32)	53.43(52.28–54.56)	53.71(52.57–54.84)	55.17(54.2–56.14)	54.60(53.48–55.71)	55.74(54.83–56.65)
Small Metropolitan	30.21(29.03–31.43)	30.11(29.12–31.12)	30.44(29.27–31.65)	30.39(29.19–31.63)	31.29(30.04–32.57)	30.2(28.83–31.61)	30.1(28.84–31.4)	29.33(28.39–30.3)	30.14(29.07–31.22)	29.89(28.93–30.86)
Non–Metropolitan	16.43(15.7–17.19)	16.64(15.72–17.59)	16.5(15.54–17.5)	16.05(15.28–16.84)	15.55(14.69–16.45)	16.38(15.4–17.4)	16.19(15.17–17.27)	15.49(14.74–16.28)	15.26(14.59–15.96)	14.37(13.54–15.25)
Health Insurer										
Private	69.11(68.15–70.05)	69.43(68.44–70.39)	67.86(66.96–68.74)	65.85(64.82–66.86)	65.03(64.06–66)	64.59(63.6–65.57)	65.67(64.69–66.63)	66.11(65.1–67.11)	66.28(65.64–66.91)	67.17(66.45–67.87)
Medicare	6.61(6.008–7.258)	7.33(6.715–7.992)	7.01(6.532–7.528)	7.53(6.947–8.161)	8.19(7.592–8.834)	8.8(8.213–9.425)	8.21(7.546–8.929)	8.91(8.357–9.502)	8.63(8.183–9.107)	8.98(8.508–9.478)
Medicaid	5.57(5.212–5.945)	5.15(4.83–5.479)	5.72(5.354–6.097)	6.16(5.765–6.573)	6.86(6.466–7.275)	6.52(6.148–6.914)	6.92(6.546–7.32)	8.56(8.104–9.028)	10.06(9.688–10.45)	10.29(9.938–10.66)
Tricare & VA	1.55(1.367–1.759)	1.46(1.263–1.68)	1.67(1.472–1.9)	1.62(1.409–1.868)	1.84(1.574–2.154)	2.01(1.779–2.279)	1.86(1.617–2.147)	1.96(1.76–2.187)	1.87(1.687–2.071)	1.85(1.658–2.058)
Other	1.81(1.585–2.076)	1.53(1.35–1.743)	1.82(1.612–2.062)	2.01(1.756–2.304)	1.75(1.562–1.961)	2.04(1.826–2.271)	1.67(1.506–1.842)	2.12(1.952–2.295)	2.59(2.417–2.764)	2.19(2.018–2.383)
Uninsured	15.36(14.61–16.14)	15.11(14.47–15.78)	15.92(15.3–16.57)	16.83(16.26–17.41)	16.32(15.75–16.91)	16.04(15.41–16.68)	15.67(15.08–16.28)	12.34(11.9–12.8)	10.57(10.15–10.99)	9.52(9.061–10)
Receives Government Assistance										
No	85.42(84.81–86)	86.04(85.25–86.8)	84.38(83.77–84.96)	82.56(81.9–83.2)	80.6(79.89–81.29)	79.61(78.93–80.28)	79.31(78.48–80.11)	79.8(79.18–80.41)	80.27(79.71–80.81)	81.29(80.74–81.83)
Yes	14.58(14–15.19)	13.96(13.2–14.75)	15.62(15.04–16.23)	17.44(16.8–18.1)	19.4(18.71–20.11)	20.39(19.72–21.07)	20.69(19.89–21.52)	20.2(19.59–20.82)	19.73(19.19–20.29)	18.71(18.17–19.26)
Income as % of Federal Poverty Level										
<100% FPL	11.61(11.04–12.22)	11.42(10.88–12)	12.09(11.57–12.62)	12.8(12.19–13.43)	14.04(13.41–14.69)	15.9(15.31–16.52)	14.7(14.05–15.38)	15.06(14.56–15.58)	15.17(14.66–15.7)	14.53(13.99–15.09)
100–199% FPL	17.85(17.09–18.62)	18.27(17.61–18.95)	19.62(18.92–20.34)	20.86(20.06–21.68)	20.69(20–21.39)	19.22(18.53–19.93)	19.62(18.84–20.43)	19.65(18.97–20.34)	20.16(19.46–20.89)	20.14(19.51–20.78)
>= 200% FPL	70.54(69.55–71.51)	70.3(69.39–71.2)	68.29(67.32–69.24)	66.34(65.25–67.42)	65.28(64.36–66.18)	64.87(63.99–65.75)	65.68(64.61–66.73)	65.29(64.31–66.25)	64.67(63.82–65.5)	65.33(64.48–66.18)

### Substance use from 2007–16 and trends within the period

**Fig 1:**
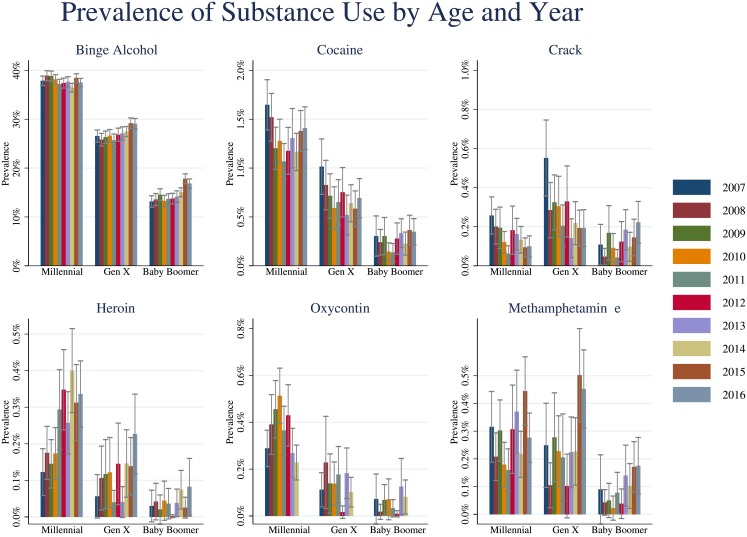
Prevalence of binge alcohol, cocaine, crack, oxycontin, heroin, and methamphetamine use by generational cohort, 2007–16. Error bars denote 95% confidence intervals.

Binge alcohol use was highest among Millennials (37.83%; 95% CI = 37.50%-38.15%) and lowest among Baby Boomers (14.66%; 95% CI = 14.33%-15.01%) with Gen X falling somewhere in between (27.04%; 95% CI = 26.64–27.45%). Use of binge alcohol varied significantly between generational cohorts from 2007–16 (*F* (2,109) = 3792.99, *p*<0.005). These patterns are shown in Fig 1 below.

Use of cocaine varied significantly between generational cohorts from 2007–16 (*F* (2,109) = 161.92, *p*<0.005) as shown in Fig 1. As with binge alcohol, use was highest among Millennials (1.31%; 95% CI = 1.24%-1.40%) and lowest among Baby Boomers (0.27%; 95% CI = 0.23%-0.32%), with Gen X (0.70%; 95% CI = 0.64%-0.77%) in the middle. In all ages, the pattern across time was either stable or reducing.

Use of crack is notably higher among Gen X (0.28%; 95% CI = 0.23%-0.33%) as compared with Millennials (0.15%; 95% CI = 0.13%-0.17%) and Baby Boomers (0.12%; 95% CI = 0.10%-0.16%), particularly in 2007 as shown in Fig 1. Heroin (*F* (2,109) = 59.11, *p*<0.005), oxycontin (*F* (2,109) = 44.34, *p*<0.005), and methamphetamine (*F* (2,109) = 26.72, *p*<0.005) all varied significantly among generational cohorts.

### Variations in substance use among Millennials vis-à-vis Gen X and Baby Boomers

The variations in past-month substance use among generational cohorts is shown in Table 2 where, generally, we observe levels of binge alcohol use among all generational cohorts ranging between 25–40% as previously mentioned and markedly low levels (<4%) of other substance use (*i*.*e*. cocaine, crack, heroin, oxycontin, and methamphetamine) among all generational cohorts, especially Baby Boomers. There is a general decline in observed substance usage with increasing age. Crack use is almost twice as much among Gen X as in Millennials. Poly-use, or the use of at least two substances excluding binge alcohol, was highest among Gen X (0.34%; 95% CI = 0.29%-0.39%), followed by Millennials (0.31%; 95% CI = 0.27%-0.34%) and lowest among Baby Boomers (0.15%; 95% CI = 0.12%-0.19%).

**Table 2:** Unadjusted and adjusted multiple logistic regression of generational cohort associated with past-month substance use and poly-use for adults, 2007–16.

	Millennial		Gen X		Baby Boomer		Unadjusted						Adjusted*					
	(*n* = 237,910)		(*n* = 88,402)		(*n* = 67,809)		Millennial		Gen X		Baby Boomer		Millennial		Gen X		Baby Boomer	
	%	95% CI	%	95% CI	%	95% CI	OR	95% CI	OR	95% CI	OR	95% CI	OR	95% CI	OR	95% CI	OR	95% CI
Binge Alcohol	37.83	(37.50–38.15)	27.04	(26.64–27.45)	14.66	(14.33–15.01)	1.64^†^	(1.60–1.68)	1.00		0.46^†^	(0.45–0.48)	1.51^†^	(1.46–1.56)	1.00		0.56^†^	(0.54–0.58)
Cocaine	1.31	(1.24–1.40)	0.70	(0.64–0.77)	0.27	(0.23–0.32)	1.88^†^	(1.68–2.12)	1.00		0.38^†^	(0.31–0.47)	1.19^†^	(1.03–1.37)	1.00		0.44^†^	(0.35–0.56)
Crack	0.15	(0.13–0.17)	0.28	(0.23–0.33)	0.12	(0.10–0.16)	0.54^†^	(0.42–0.70)	1.00		0.45^†^	(0.34–0.60)	0.33^†^	(0.25–0.43)	1.00		0.54^†^	(0.37–0.79)
Heroin	0.26	(0.23–0.29)	0.11	(0.09–0.14)	0.04	(0.03–0.06)	2.28^†^	(1.76–2.97)	1.00		0.35^†^	(0.22–0.58)	1.39^†^	(1.01–1.91)	1.00		0.37^†^	(0.21–0.64)
Oxycontin	0.37	(0.33–0.41)	0.14	(0.10–0.18)	0.06	(0.04–0.09)	2.68	(1.98–3.62)	1.00		0.44	(0.27–0.72)	2.33^†^	(1.74–3.11)	1.00		0.42	(0.24–0.72)
Methamphetamine	0.28	(0.24–0.32)	0.26	(0.22–0.30)	0.09	(0.07–0.12)	1.09	(0.89–1.34)	1.00		0.36^†^	(0.27–0.49)	0.89	(0.67–1.18)	1.00		0.36^†^	(0.26–0.52)
Poly–Use	0.31	(0.27–0.34)	0.34	(0.29–0.39)	0.15	(0.12–0.19)	0.90	(0.73–1.10)	1.00		0.44	(0.33–0.58)	0.56	(0.45–0.70)	1.00		0.50	(0.36–0.71)

*Adjusted for year, sex, ethnicity, level of education, marital status, self reported health, metropolitan size, type of health insurance, whether the respondent was receiving government assistance, income, and income as a percentage of the federal poverty limit (FPL)

^†^Denotes statistical significance (*p*<0.05)

The results of unadjusted and adjusted multivariable logistic regression of substance use and generational cohort are also shown in Table 2. Controlling for year, socioeconomic, demographic, and health covariates, Millennials were at significantly increased risk of use of binge alcohol (AOR = 1.51; 95% CI = 1.46–1.56) and oxycontin (AOR = 2.33; 95% CI = 1.74–3.12) than Gen X while Baby Boomers were at significantly reduced risk of all substances: binge alcohol (AOR = 0.56; 95% CI = 0.54–0.58), cocaine (AOR = 0.44; 95% CI = 0.35–0.56), crack (AOR = 0.54; 95% CI = 0.37–0.79), heroin (AOR = 0.37; 95% CI = 0.21–0.64), oxycontin (AOR = 0.42; 95% CI = 0.24–0.72), methamphetamine (AOR = 0.36; 95% CI = 0.26–0.52), and poly-use (AOR = 0.37; 95% CI = 0.30–0.47). Millennials were at significantly reduced risk of crack use (AOR = 0.33; 95% CI = 0.25–0.43) and poly-use (AOR = 0.56; 95% CI = 0.45–0.70)as compared to Gen X.

### Variations in substance use and vulnerability

**Fig 2:**
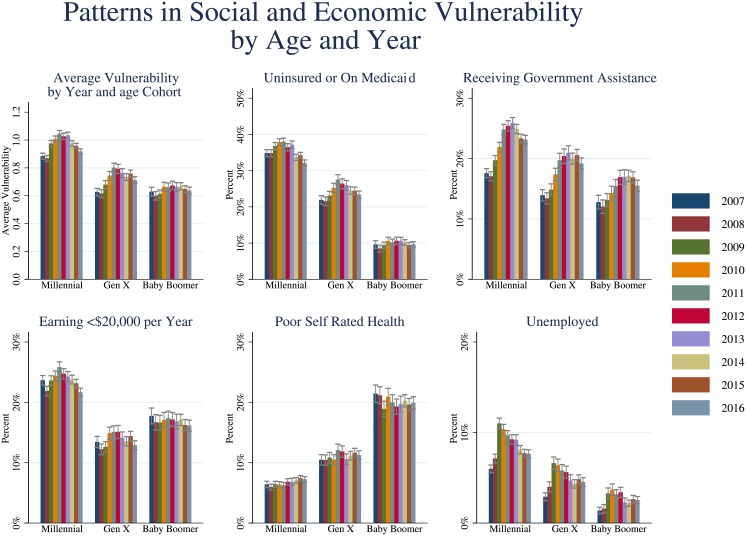
Patterns in social and economic vulnerability by generational cohort, 2007–16. Error bars denote 95% confidence intervals.

The range of vulnerability in the study population is shown in Fig 2. Average vulnerability was highest among Millennials (0.97; 95% CI = 0.96–0.98) and lowest among Baby Boomers (0.65; 95% CI = 0.64–0.66) with Gen X in the middle (0.72; 95% CI = 0.71–0.73). As shown in Table 3, increasing vulnerability is associated with increased risk of cocaine, crack, heroin, oxycontin, and methamphetamine with particularly pronounced effects on crack, heroin, and methamphetamine use. For instance, for crack use, those with a vulnerability score of 1 exhibit an adjusted odds ratio of 3.96 (95% CI = 2.49–6.30) while those with a vulnerability score of 5 exhibit an adjusted odds ratio of 55.32 (24.63–124.28) with a trend of increasing odds of crack use with increasing vulnerability.

**Table 3:** Adjusted multiple logistic regression of vulnerability associated with past-month substance use and poly-use for adults, 2007–16.

	Vulnerability												Adjusted*											
	0		1		2		3		4		5													
	(*n* = 187,749)		(*n* = 102,149)		(*n* = 56,507)		(*n* = 35,194)		(*n* = 11,586)		(*n* = 936)		0		1		2		3		4		5	
	%	95% CI	%	95% CI	%	95% CI	%	95% CI	%	95% CI	%	95% CI	OR	95% CI	OR	95% CI	OR	95% CI	OR	95% CI	OR	95% CI	OR	95% CI
Binge Alcohol	24.67	(24.38–24.97)	25.81	(25.30–26.33)	25.03	(24.36–25.71)	24.07	(23.31–24.85)	26.86	(25.46–28.32)	31.96	(27.76–36.48)	1.00		0.99	(0.96–1.02)	0.91^†^	(0.88–0.95)	0.89^†^	(0.85–0.93)	1.07	(0.99–1.16)	1.23	(0.99–1.53)
Cocaine	0.40	(0.3721–0.432)	0.81	(0.7379–0.8915)	1.15	(1.009–1.307)	1.51	(1.312–1.748)	2.19	(1.857–2.569)	2.91	(0.6662–0.74)	1.00		1.90^†^	(1.69–2.13)	2.65^†^	(2.26–3.11)	3.63^†^	(3.04–4.33)	5.4^†^	(4.49–6.48)	6.50^†^	(3.56–11.86)
Crack	0.04	(0.0288–0.0574)	0.15	(0.1134–0.1862)	0.38	(0.3063–0.4773)	0.64	(0.502–0.8098)	1.12	(0.8546–1.454)	2.30	(0.1552–0.1922)	1.00		3.96^†^	(2.49–6.3)	10.45^†^	(6.75–16.18)	17.42^†^	(10.68–28.42)	28.68^†^	(17.4–47.27)	55.32^†^	(24.63–124.28)
Heroin	0.04	(0.0331–0.0492)	0.11	(0.0891–0.1347)	0.28	(0.2341–0.3441)	0.47	(0.3699–0.5844)	0.45	(0.3255–0.6303)	2.07	(0.1143–0.1388)	1.00		2.65^†^	(2.01–3.48)	6.88^†^	(5.1–9.28)	11.58^†^	(8.48–15.82)	11.42^†^	(7.55–17.28)	47.29^†^	(21.22–105.39)
Oxycontin	0.09	(0.0718–0.1249)	0.18	(0.1495–0.2239)	0.33	(0.2599–0.4109)	0.37	(0.2813–0.4781)	0.69	(0.4522–1.065)	0.29	(0.1549–0.1968)	1.00		1.92^†^	(1.34–2.75)	3.74^†^	(2.57–5.42)	4.60^†^	(3.14–6.73)	9.35^†^	(5.38–16.26)	3.33^†^	(1.01–10.91)
Methamphet–amine	0.07	(0.0526–0.0838)	0.19	(0.1534–0.229)	0.50	(0.4114–0.6177)	0.49	(0.3996–0.5993)	0.78	(0.5487–1.121)	1.73	(0.1735–0.2145)	1.00		3.04^†^	(2.21–4.17)	8.56^†^	(6.24–11.72)	8.69^†^	(6.15–12.28)	14.46^†^	(8.89–23.54)	28.91^†^	(13.41–62.33)
Poly–Use	0.08	(0.06–0.10)	0.20	(0.16–0.24)	0.58	(0.48–0.69)	0.83	(0.68–1.01)	1.35	(1.06–1.72)	2.90	(1.61–5.17)	1.00		2.78^†^	(2.01–3.84)	8.19	(5.94–11.29)	12.02	(8.43–17.14)	19.02	(13.02–27.80)	37.74	(18.93–75.24)

*Adjusted for sex, ethnicity, and generational cohort

^†^Denotes statistical significance (*p*<0.05)

**Fig 3:**
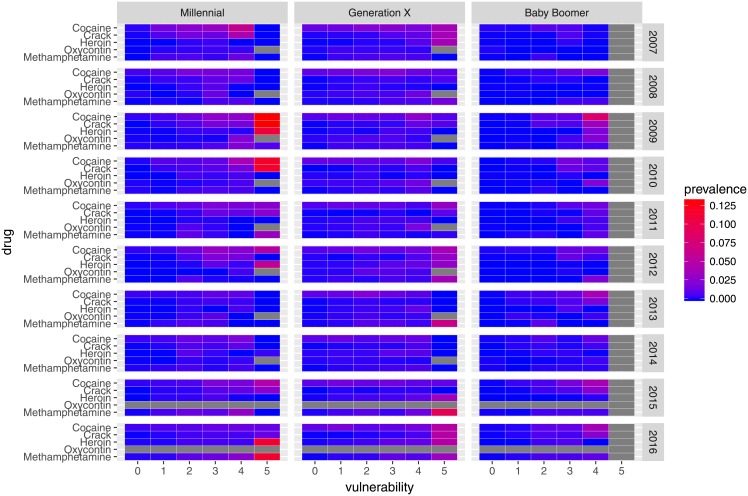
Patterns in prevalence of substance use by generational cohort and vulnerability, 2007–16. Grey cells denote missing data.

As shown in Fig 3, we observe some patterns of substance use associated with generational cohort and vulnerability by year. The highest prevalence of cocaine, crack, heroin, and poly-use was observed among individuals of highest vulnerability from the Generation X cohort during 2009. Among Millennials, use of methamphetamine and poly-use was highest in 2013. For all three generational cohorts, increasing vulnerability appears to be linked with increased prevalence of poly-use.

### Other social, economic, and health correlates of substance use

Table 4 shows the results of multivariable logistic regression for past-month use of any substance against other socioeconomic, demographic, and health variables in this study. Women were at significantly reduced risk of substance use in general (AOR = 0.48; 95% CI = 0.43–0.52) as were those of Asian & Pacific Islander descent (AOR = 0.34; 95% CI = 0.25–0.46). Those with either secondary (AOR = 1.89; 95% CI = 1.30–2.74) or tertiary education (AOR = 1.92; 95% CI = 1.32–2.81) were more at risk of substance use than those with only an elementary education. Those making over $20,000 a year were all at reduced risk of substance use. Individuals who were married (AOR = 0.30; 95% CI = 0.25–0.35) or widowed (AOR = 0.36; 95% CI = 0.22–0.57) were at less risk of substance use than single individuals.

**Table 4:** Adjusted multiple logistic regression of socioeconomic, demographic, and health variables associated with past-month substance use and any-use for adults, 2007–16.

	Any Past–Month Substance Use				Adjusted*	
	No		Yes			
	(*n* = 303,311; 76.96%)		(*n* = 90,810; 23.04%)			
	%	95% CI	%	95% CI	OR	95% CI
Sex						
Male	98.59	(98.50–98.67)	1.41	(1.33–1.50)	1.00	
Female	99.33	(99.28–99.38)	0.67	(0.62–0.72)	0.48^†^	(0.43–0.52)
Ethnicity						
White	98.95	(98.89–99.01)	1.05	(0.99–1.11)	1.00	
Black	98.88	(98.73–99.01)	1.12	(0.99–1.28)	0.57^†^	(0.49–0.67)
Native American	99.00	(98.87–99.11)	1.00	(0.89–1.13)	0.56^†^	(0.48–0.64)
Asian & Pacific Islander	99.59	(99.45–99.69)	0.41	(0.31–0.55)	0.34^†^	(0.25–0.46)
Hispanic	98.41	(97.81–98.84)	1.60	(1.16–2.19)	0.92^†^	(0.66–1.29)
Other	98.39	(98.03–98.68)	1.61	(1.32–1.97)	0.98	(0.79–1.20)
Employment						
Full–Time	99.10	(99.04–99.16)	0.90	(0.84–0.96)	1.00	
Part–Time	98.78	(98.66–98.90)	1.22	(1.10–1.34)	1.14^†^	(1.01–1.29)
Unemployed	97.14	(96.85–97.41)	2.86	(2.59–3.16)	1.56^†^	(1.37–1.78)
Other (including not in labor force)	99.15	(99.07–99.23)	0.85	(0.77–0.93)	1.11	(0.97–1.26)
Education						
Elementary	99.49	(99.27–99.64)	0.51	(0.36–0.73)	1.00	
Secondary	98.71	(98.64–97.78)	1.29	(1.22–1.36)	1.89^†^	(1.30–2.74)
Tertiary	99.13	(99.08–99.19)	0.87	(0.81–0.92)	1.92^†^	(1.32–2.81)
Income						
Less than 20,000	98.13	(97.97–98.27)	1.87	(1.73–2.03)	1.00	
20,000 to 49,999	98.94	(98.85–99.01)	1.07	(0.99–1.15)	0.64^†^	(0.53–1.29)
50,000 to 74,999	99.19	(99.09–9928)	0.81	(0.72–0.91)	0.60^†^	(0.46–0.78)
More than 75,000	99.37	(99.30–99.43)	0.63	(0.57–0.70)	0.58^†^	(0.46–0.74)
Marital Status						
Single	97.72	(97.62–97.83)	2.28	(2.17–2.38)	1.00	
Married	99.64	(99.59–99.68)	0.36	(0.32–0.41)	0.30^†^	(0.25–0.35)
Widowed	99.69	(99.53–99.80)	0.31	(0.20–0.47)	0.36^†^	(0.22–0.57)
Divorced or Separated	98.54	(98.37–98.69)	1.46	(1.31–1.63)	0.94	(0.82–1.08)
Self Rated Health						
Excellent	99.36	(99.29–99.42)	0.64	(0.58–0.71)	1.00	
Very Good	99.06	(99.00–99.12)	0.94	(0.88–1.01)	1.50^†^	(1.33–1.69)
Good	98.82	(98.72–98.90)	1.18	(1.10–1.28)	1.94^†^	(1.71–2.21)
Poor	98.43	(98.26–98.59)	1.57	(1.41–1.74)	2.86^†^	(2.41–3.38)
Metropolitan Statistical Area Size						
Large Metropolitan	98.89	(98.82–98.96)	1.11	(1.04–1.18)	1.00	
Small Metropolitan	98.98	(98.90–99.05)	1.03	(0.95–1.10)	0.84^†^	(0.76–0.92)
Non–Metropolitan	99.26	(99.16–99.35)	0.74	(0.65–0.84)	0.56^†^	(0.48–0.64)
Health Insurer						
Private	99.36	(99.32–99.40)	0.64	(0.60–0.68)	1.00	
Medicare	99.23	(99.02–99.40)	0.77	(0.60–0.98)	1.21	(0.94–1.56)
Medicaid	97.89	(97.67–98.10)	2.11	(1.90–2.33)	1.61^†^	(1.38–1.87)
Tricare & VA	98.87	(98.47–99.16)	1.13	(0.84–1.53)	1.45^†^	(1.06–1.98)
Other	98.32	(97.88–98.67)	1.68	(1.33–2.13)	1.57^†^	(1.22–2.01)
Uninsured	97.68	(97.49–97.85)	2.32	(2.15–2.51)	1.94^†^	(1.74–2.17)
Receives Government Assistance						
No	99.17	(99.13–99.21)	0.83	(0.79–0.87)	1.00	
Yes	98.08	(97.83–98.22)	1.92	(1.78–2.07)	1.46^†^	(1.32–1.63)
Income as % of Federal Poverty Level						
<100% FPL	98.18	(98.03–98.31)	1.82	(1.69–1.97)	1.00	
100–199% FPL	98.70	(98.58–98.82)	1.30	(1.18–1.42)	1.26^†^	(1.09–1.45)
>= 200% FPL	99.22	(99.17–99.26)	0.78	(0.98–1.07)	1.58^†^	(1.29–1.93)

*Adjusted for year, sex, ethnicity, generational cohort, level of education, marital status, self reported health, metropolitan size, type of health insurance, whether the respondent was receiving government assistance, income, and income as a percentage of the federal poverty limit (FPL)

^†^Denotes statistical significance (*p*<0.05)

**Fig 4:**
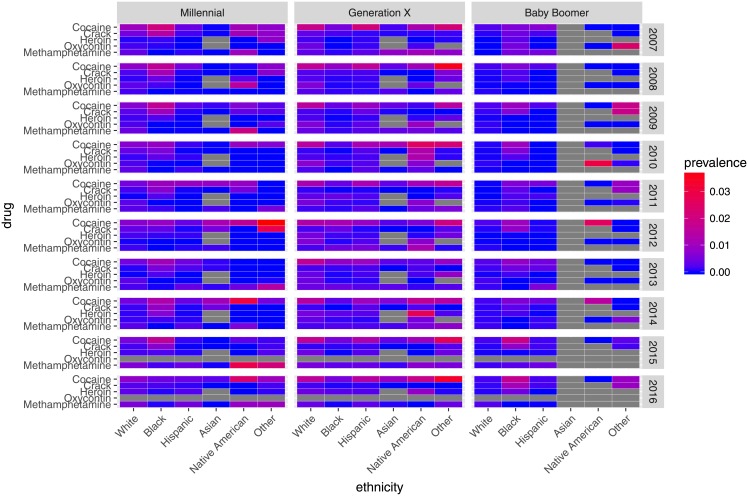
Patterns in prevalence of substance use by generational cohort and ethnicity, 2007–16. Grey cells denote missing data.

Fig 4 depicts patterns of substance use by generational cohort and ethnicity from 2007–16. Native Americans among the Millennial cohort exhibited the greatest prevalence of poly-use in 2014. Substance use appears higher among Millennials, primarily White and Native American Millennials, than other generational cohorts. Among Generation X, substance use appears highest among those identifying as Native American or Other, particularly in 2012 for cocaine, crack, and poly-use, and in 2014 for poly-use.

## Discussion

Existing knowledge regarding the effects of economic recessions, particularly the GFC, and health have previously been linked to worsening in mental health, reductions in road traffic deaths, and short-term associations with cancer and heart disease [31]. Our analysis extends this by highlighting major differences in binge alcohol and substance use, including poly-use, between Millennials and other generational cohorts. Millennials exhibited statistically significant higher risk of substance use over the study period, 2007–16, with especially worrisome patterns of increase in heroin and oxycontin use, a trend which is particularly concerning in the context of the recent prescription opioid and heroin epidemic [32] and the measures introduced by the Obama administration to address this crisis such as improved access to naloxone and improved training among law enforcement agencies [33]. These differences appear to be exacerbated by the impact of austerity and a high degree of socioeconomic vulnerability, including: being uninsured or insured on Medicaid, receiving government assistance, income less than 100% FPL, poor self rated health, and unemployment. Increased social and economic vulnerability after the 2007 crisis is associated significantly with higher rates of substance use.

A limitation of this study is the use of survey data to ascertain both socioeconomic and demographic characteristics of the study population as well as the use of self reported substance use as an outcome variable. As we are unable to ascertain specific birth years for individuals, we infer generational cohort membership based on a combination of self reported age and year of data collection. Moreover, underreporting of substance use may affect the accuracy of prevalence estimates for past-month binge alcohol and substance use as no objective or clinical measure of substance use was collected with the survey. Moreover, small samples for specific subpopulations in this study, such as Asian & Pacific Islander, those with an elementary education level or less, or those insured by Tricare & VA, limit our ability to detect specific patterns of substance use in these populations. The design of the NSDUH as a household survey, moreover, does not permit for the ability to sample homeless or institutionalised persons who may exhibit markedly different patterns of substance use. Finally, the use of repeated, cross-sectional data does not allow for an assessment of individuals over time and, consequently, no causality can be established between the outcome variables and the exposures of interest. Despite these limitations, the NSDUH remains a robust source of epidemiological data for assessing the prevalence of substance use. It has been shown to provide comparable findings to other validated health studies such as the National Comorbidity Survey Replication (NCS-R) and remains the only survey in the United States which provides nationally representative statistics on substance use over the life course, from adolescence through to adulthood [34]. Its relatively large annual sample size (>65,000), deeply stratified sampling design to ensure representation among and within states, and face-to-face modality further highlight its strengths as a source of data for our analyses [25].

Our analyses highlight how a major macroeconomic downturn, the GFC, is associated with disproportionately adverse outcomes among Millennials vs. Gen X and Baby Boomers with respect to binge alcohol and substance use. We highlight several social, health, and economic correlates of substance use and highlight the link between social and economic vulnerability and substance use. These findings suggest a need for greater attention towards the ways in which macroeconomy and population health are linked and how these effects may disproportionately affect generational subpopulations with implications for health equity, health services provision, and policymaking. A growing body of literature has emerged, for instance, documenting the efficacy of differential health interventions to alleviate binge alcohol use among young [35] and older [36] adults; comparable work to identify the unique needs of socioeconomically disadvantaged Millennials vis-à-vis advantaged Baby Boomers with respect to binge alcohol and substance use to help address ongoing problems such as the ongoing prescription opioid crisis. Further work may elucidate the relative merits of a harm reduction approach for Millennials, for example, rather than abstinence. These findings also signal a need to address economic inequalities with persist between Millennials and older generational cohorts while alleviating the direct causes of vulnerability, chiefly caused by weak macroeconomic conditions, poor career prospects despite high educational attainment, and government policies which have exacerbated austerity and inequality.

Existing economic research has shown that demand for some substances, such as cocaine, heroin, and methamphetamine is highly elastic which can help explain, in part, some characteristics of first-time substance users, depending on macroeconomic conditions and prices of substances [23]. As income decreases, particularly in times of economic hardship, we would expect substance use to decline and so our findings are consistent with this earlier research. Nevertheless, more research is needed to better characterize the ways in which macroeconomy and first-time substance use or increase rate of consumption are linked.

Our results signal the urgent need for much greater attention to the link between intergenerational inequality and population health, particularly in a global macroeconomic climate recovering from the GFC. While governments have sought to apply monetary levers to alleviate the worst effects of this global recession, our findings underscore the fact that Millennials have suffered disproportionately, exhibiting increased socioeconomic vulnerability and increased use of binge alcohol and substance use as compared to Gen X and Baby Boomers. Though the long term effects of sustained socioeconomic vulnerability and intergenerational inequality have yet to manifest, policymakers should devote greater attention and sensitivity to the ways in which public policy can be further levered to ameliorate the social, economic, and health condition of Millennials.

Research is also needed to consider whether the findings presented here are consistent among other developed countries, such as the United Kingdom, to both characterize the ways in which macroeconomy, intergenerational inequality, and binge alcohol & substance use are related. In addition, given the different economic policies that countries have used to addressed the impacts of the GFC and the varying severity with which the crisis affected individual countries, there is an opportunity to directly observe the relationship between the severity of macroeconomic shocks and measures of population health such as substance use and mental health.
